# The risk of cannabis use disorder is mediated by altered brain connectivity: A chronnectome study

**DOI:** 10.1111/adb.13395

**Published:** 2024-05-06

**Authors:** Giovanni Fazio, Daniele Olivo, Nadine D. Wolf, Dusan Hirjak, Mike M. Schmitgen, Florian Werler, Miriam Witteman, Katharina M. Kubera, Vince D. Calhoun, Wolfgang Reith, Robert Christian Wolf, Fabio Sambataro

**Affiliations:** ^1^ Department of Neuroscience, Padua Neuroscience Center University of Padua Padua Italy; ^2^ Department of General Psychiatry at the Center for Psychosocial Medicine Heidelberg University Heidelberg Germany; ^3^ Department of Psychiatry and Psychotherapy, Central Institute of Mental Health, Medical Faculty Mannheim Heidelberg University Mannheim Germany; ^4^ Department of Psychiatry and Psychotherapy Saarland University Saarbrücken Germany; ^5^ Tri‐institutional Center for Translational Research in Neuroimaging and Data Science (TReNDS), Georgia State University, Georgia Institute of Technology Emory University Atlanta Georgia USA; ^6^ Department of Neuroradiology Saarland University Saarbrücken Germany

**Keywords:** cannabis, chronnectome, dynamic connectivity, magnetic resonance imaging, meta‐states, resting state

## Abstract

The brain mechanisms underlying the risk of cannabis use disorder (CUD) are poorly understood. Several studies have reported changes in functional connectivity (FC) in CUD, although none have focused on the study of time‐varying patterns of FC. To fill this important gap of knowledge, 39 individuals at risk for CUD and 55 controls, stratified by their score on a self‐screening questionnaire for cannabis‐related problems (CUDIT‐R), underwent resting‐state functional magnetic resonance imaging. Dynamic functional connectivity (dFNC) was estimated using independent component analysis, sliding‐time window correlations, cluster states and meta‐state indices of global dynamics and were compared among groups. At‐risk individuals stayed longer in a cluster state with higher within and reduced between network dFNC for the subcortical, sensory‐motor, visual, cognitive‐control and default‐mode networks, relative to controls. More globally, at‐risk individuals had a greater number of meta‐states and transitions between them and a longer state span and total distance between meta‐states in the state space. Our findings suggest that the risk of CUD is associated with an increased dynamic fluidity and dynamic range of FC. This may result in altered stability and engagement of the brain networks, which can ultimately translate into altered cortical and subcortical function conveying CUD risk. Identifying these changes in brain function can pave the way for early pharmacological and neurostimulation treatment of CUD, as much as they could facilitate the stratification of high‐risk individuals.

## Graphical abstract

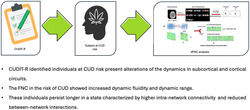

## 1 INTRODUCTION

Cannabis is the third most widely used controlled substance in the world, after alcohol and tobacco, with estimates from the United Nations dating back to 2020, which indicate that 209 million people have used cannabis at least once in the previous year.^1^ Among 1 in 10 regular users and 1 in 3 individuals using this substance daily^2^, ^3^ develop a problematic use with an inability to stop using cannabis despite clinical and psychosocial problems, that is, Cannabis Use Disorder (CUD).^4^

CUD has been associated with an increased risk of developing concomitant psychiatric disorders and contributes to the evolution of psychotic disorders in ultra‐high risk subjects.^5^ In this regard, observational and experimental studies have confirmed the association between cannabis consume and the onset and persistence of psychotic disorders with an effect size that is related to the duration of abuse and to the potency of cannabis.^6^ In particular, cannabis use was linked with an earlier onset of psychosis, greater symptom severity, higher rates of relapse, longer hospitalizations and poorer outcomes.^7^ Furthermore, chronic use of cannabis was also associated with a worse prognosis, including treatment resistance for various psychiatric disorders^8^ and a greater risk of manic episodes and suicide.^6^

Several factors can contribute to the development of CUD in at‐risk individuals. These include genetic and psychological factors (e.g., emotional dysregulation), comorbid mental disorders (including depression, anxiety, attention deficit hyperactivity disorder [ADHD], conduct and/or personality disorders), pattern of cannabis use (frequency and duration of use, concomitant use of alcohol and tobacco and other substances), parental cannabis use and stressful life events (childhood abuse, unemployment, financial difficulties).^9^

At the biological level, Koob and Volkow^10^ propose a more general model of substance addiction that can be applied to cannabis addiction^11^ and encompasses three stages: binge/intoxication, withdrawal/negative affect and preoccupation/anticipation. The first entails excessive impulsive and compulsive behaviours aimed at consuming cannabis despite the negative consequences associated with its use. This stage is characterized by hyperactivation of the mesocorticolimbic dopaminergic reward pathway caused by an impairment in incentive salience, which appears to drive dopaminergic signalling to maintain the drug consume upon exposure to conditioned cues and during tolerance development. These continuous mechanisms of opponent‐process responses trigger the withdrawal/negative affect stage. This stage is characterized by neurobiological changes that take place within‐systems, including decreased dopaminergic signalling in the nucleus accumbens (NAcc) and dorsal striatum that result in an elevation of reward thresholds for non‐drug reinforcers, and between‐systems, which include changes in the stress responses of the brain, including corticotropin‐releasing factor (CRF) release in the amygdala and HPA‐axis dysfunction. These changes can lead to loss of motivation for non‐drug rewards and impaired emotion regulation during acute and sustained abstinence. The last stage, preoccupation/anticipation, is involved in the re‐establishment of substance use after abstinence. At the neural level, the GABAergic and glutamatergic signalling pathways between the pre‐frontal cortex (PFC) and the brain areas mediating decision‐making, self‐regulation, inhibitory control and working memory are altered. At the behavioural level, this stage may be associated with excessive salience attribution to drug‐paired cues, decreases in responsiveness to non‐drug cues and reinforcers and reduction of the ability to inhibit maladaptive behaviour.^10^, ^11^ Overall, the neural changes implicated in vulnerability and the risk of developing CUD need to be further explored.

Neuroimaging studies have shown that the brain is intrinsically organized in functionally connected networks that interact with each other to determine a specific function and their spontaneous activity can be studied using resting state functional Magnetic Resonance Imaging (rs‐fMRI) rather than using specific tasks that engage few regions and can be influenced by performance.^12^ Numerous studies in fMRI have shown that cannabis use can alter brain functioning, especially in networks that support working memory, attention and cognitive control processing.^13^ More specifically, a recent systematic review found that a substantial number of studies (40%) conducted in cannabis users showed a higher positive resting state FC in fronto‐frontal, fronto‐temporal and fronto‐striatal networks relative to controls.^14^ Moreover, alterations of functional connectivity (FC) at rest have been reported in individuals with CUD relative to healthy controls, although their direction has shown mixed.^15^ These contrasting findings are due in part to the temporal characteristics of FC. Indeed, FC cannot be considered stationary but varies over time with functional coupling between regions and networks that dynamically rearrange in various connectivity modes.^16^ For this reason, the use of a static analysis approach may oversimplify a model that eventually fails to capture the complexity of brain FC.

Recently, to overcome these limitations, a new analytic framework, dFNC, that investigates the temporal changes of brain connectivity over time (and, for this reason, defined as ‘chronnectome’) has been developed.^16^, ^17^, ^18^, ^19^, ^20^, ^21^ DFNC studies the recurring patterns of FC between different functional brain networks, namely, FC ‘states’, that are reproducible over time and between different subjects.^17^, ^22^, ^23^

DFNC can be studied with two different and complementary methods: a cluster‐state approach and a meta‐state approach. The first is based on a deterministic assumption, with a subject dwelling in only a single state of connectivity at a given point in time, and reveals pairwise network connectivity patterns arranged in states and the changes in their temporal engagement.^16^ The second arises from a probabilistic view of the connectivity states where a subject can occupy several states of connectivity at each time, ‘meta‐states’,^24^ and provides estimates of the overall dynamics of FC between states with metrics of dFNC fluidity and range.^16^, ^24^, ^25^, ^26^, ^27^

In this study, our major aim was to understand the role of altered dFNC in the development of CUD. For this, we wanted to identify alterations in dFNC in individuals at risk of CUD. Moreover, to confirm the possible role of these neural changes in the clinical presentation of CUD, we correlated dFNC measures with clinical variables in cannabis users. Notably, we recruited only individuals at risk of cannabis addiction: first, to understand the mechanisms associated with the development of the CUD and, second, to study the effects of cannabis use while reducing the confounding effects of clinical and psychosocial changes after the problematic use of cannabis.^28^, ^29^, ^30^, ^31^

## 2 MATERIALS AND METHODS

### 2.1 Participants and MRI data

Ninety‐eight subjects were initially recruited for this study. Exclusion criteria were considered: a present or past mental disorder assessed using the structured clinical interview (SCID) for the DSM‐IV‐TR; current or past substance use other than cannabis and nicotine; and history of neurological disorders, brain trauma or other concurrent drug treatment. None of the participants met the diagnostic criteria for the DSM‐IV‐TR cannabis use disorder. After recruitment, four participants were excluded for not having undergone an imaging session (*n* = 3) or clinical data appointment (*n* = 1). A final sample of 94 individuals (66 males and 28 females, aged between 18 and 30 years) was included in the study.

The following demographic data were collected: age, sex, handedness and education. All participants were also evaluated for depression, anxiety and ADHD using Hamilton Depression (HAM‐D),^32^, ^33^ the State and Trait Anxiety Inventory (STAI‐G X1, STAI‐G X2)^34^ and the German adult ADHD symptoms self‐rated scale (Aufmerksamkeitsdefizit‐/Hyperaktivitätsstörung im Erwachsenenalter, Selbstbeurteilungsskala, ADHS‐SB).^35^

Participants were stratified according to their risk of developing CUD using a screening tool, the Cannabis Use Disorder Identification Test (CUDIT‐R), with the risk of CUD defined by a score greater than or equal to 8 as reported previously,^36^, ^37^ thus resulting in 55 controls and 39 individuals at risk of CUD. Patterns of cannabis use (lifetime total cannabis use, age of onset, duration and current use [days/week and g/week]) were used to evaluate cannabis use (see Table 1).

**TABLE 1:** Demographics, substance use characteristics and psychometric scores.

	Control s *n* = 55 Mean ± SD	CUD at‐risk *n* = 39 Mean ± SD	*p* ^a^
Age (years)	23.30 ± 3	23.20 ± 3.31	0.77
Sex (M:F)	30:25	36:3	0.006
Handedness (R:L)	52:3	36:3	0.202
Education (years)	14.90 ± 2.68	13.70 ± 2.57	0.033
HAM‐D	0.364 ± 0.729	1.38 ± 2.22	0.002
STAI‐G X1	31.00 ± 7.47	36.30 ± 8.03	0.001
STAI‐G X2	31.50 ± 7.74	35.60 ± 8.75	0.018
ADHD‐SB tot	6.110 ± 5.99	13.10 ± 6.94	<0.001
ADHS inattentive	2.98 ± 2.55	5.82 ± 3.10	<0.001
ADHS impulsivity	0.836 ± 1.13	2.18 ± 2.14	<0.001
ADHS hyperactivity	1.62 ± 2.21	3.03 ± 2.28	0.003
Tobacco per year (pack/year)	0.942 ± 3.090	2.50 ± 4.65	0.054
N. joints lifetime	2000 ± 8,430	3,258 ± 3,949	<0.001
CUDIT‐R total	1.040 ± 2.04	16.90 ± 7.64	<0.001
Age of the first consume (years)	4.670 ± 8.17	17.70 ± 2.89	<0.001
Duration use (years)	0.373 ± 1.57	3.41 ± 3.39	<0.001
Current use (days/week)	0.473 ± 1.41	4.95 ± 2.32	<0.001
Current use (g/week)	0.212 ± 0.779	5.01 ± 5.53	<0.001

*Note*: Significant results are emboldened.

Abbreviations: ADHS‐SB, ADHS‐Selbstbeurteilungsskala; CUD, Cannabis use disorder; HAM‐D, Hamilton Rating Scale for Depression; STAI‐G X1, State–Trait Anxiety Inventory German version Form X1; STAI‐G X2, State–Trait Anxiety Inventory German version Form X2.

^a^ Results from two‐sample *t* tests or Chi squared tests, when appropriate.

Consumers were asked to abstain from cannabis use for at least 24 h before clinical assessment and MRI. Participants consented to these study‐specific requirements, and no craving or other withdrawal symptoms were reported before the MRI scanning. The study was carried out in accordance with the Declaration of Helsinki, and the protocol was approved by the ethical review board of the Saarland Medical Association, Saarbrücken, Germany. Written informed consent was obtained from all participants after the study procedures had been fully explained.^38^

### 2.2 MRI data acquisition

The scans were obtained using a 3T Magnetom‐type Skyra (Siemens, Erlangen, Germany) MRI system. Images in structural MRI were acquired through rapid sequences prepared at gradient‐echo magnetization (3D‐MPRAGE) set with the following parameters: TE = 3.29 ms, TR = 1900 ms, TI = 110 ms, flip angle = 9°, FOV = 240 mm, slice plane = axial, voxel size = 0.5 × 0.5 × 0.9 mm^3^, distance factor = 50%, number of slices = 192.

Then, rs‐fMRI images were obtained using a BOLD echo‐planar (EPI) sequence set with the following parameters: TE = 30 ms, TR = 1800 ms, flip angle = 90°, FOV = 192 mm, slice plane = transversal, voxel size = 3 × 3 × 3 mm, distance factor = 25%, number of slices = 32, PAT factor = 2, number of measurements = 230.

### 2.3 MRI data analysis

After discarding the first eight scans, acquired to allow the signal to reach the steady state, 222 volumes were pre‐processed.

Data Processing Assistant for rs‐fMRI (DPABI/DPARSF 5.2) was used to pre‐process the neuroimaging data.^39^ A slice‐timing correction was used to correct for temporal mismatch in slice acquisition. Next, realignment was used to correct head movements through rigid‐body transformations and estimate motion extent. Functional and structural images were coregistered, segmented and normalized on a Montreal Neurological Institute (MNI) template using DARTEL.^40^ Finally, a 6‐mm 3D Gaussian kernel full width at half maximum was used for smoothing.

The data were decomposed into functional networks using a spatial group‐independent component analysis (ICA),^41^ implemented through the group ICA of fMRI toolbox (GIFT)^42^ (https://trendscenter.org/software/gift/). The group information guided ICA (GIG‐ICA) algorithm was used for constrained ICA on the NeuroMark template, which comprises 53 intrinsic connectivity networks (ICNs) that were estimated in two large independent samples and span across seven functional network domains: subcortical (SC), auditory (AUD), sensorimotor (SM), visual (VIS), cognitive‐control (CC), default mode (DM) and cerebellar (CB).^43^ To increase the reliability and stability of the estimation, the ICA was repeated 50 times using the ICASSO toolbox.^44^, ^45^

### 2.4 Dynamic functional network connectivity

**FIGURE 1:**
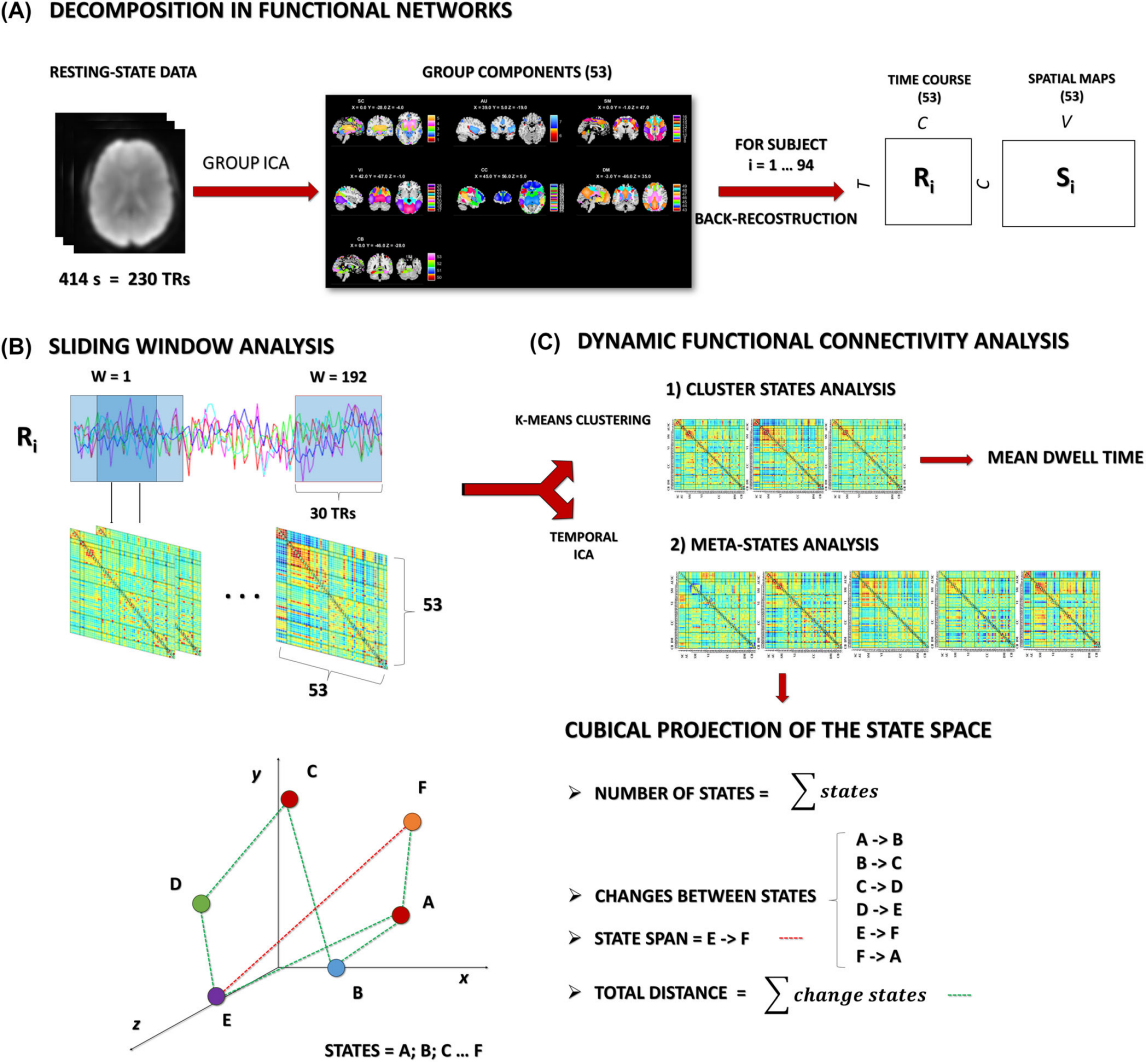
Graphical representation of the dFNC analysis process. (A) Resting‐state data were decomposed using a spatial group ICA, thus identifying 53 group components according to the NeuroMark template, each composed of a time course (Ri) and a spatial map (Si). These components were then back‐reconstructed at the subject level i. (B) A sliding window analysis was performed on the time courses. A window of 30 repetition times (TR) moved over the time course of 1 TR at a time, resulting in 192 windows. On each window a 53‐by‐53 functional connectivity matrix (wFNC) was calculated. (C) On the total of wFNCs: (1) a k‐means clustering was used to identify the cluster states and the mean dwell time for each of them (the average time a subject stayed in the same state); (2) a temporal ICA was used to identify temporally independent components, the meta‐states that spanned across a five‐dimensional space and were described using several measures indicated on the bottom right of the figure. For display purposes, we displayed the metastate measures in a three‐dimensional space (x, y, z). Each state is represented by a dot, and the path from one state to another is displayed using a dotted line. The number of states is calculated as the total of number of dots; the change between states is represented by the length of the green lines connecting two dots; the state span (red line) is the maximum distance between dots; the total distance is the sum of the length of each green line.

DFNC was estimated using the GIFT toolbox, through sliding window analysis.^22^, ^46^ For each subject, the 53 (one for each ICN) time‐courses (TC) were detrended, orthogonalized with respect to 12‐motion parameters, despiked using 3dDespike (https://afni.nimh.nih.gov/pub/dist/doc/program_help/3dDespike.html) and, subsequently, filtered with a fifth‐order Butterworth low‐pass filter (cut‐off of 0.15 Hz).^47^ A sparse matrix of inverse covariance was calculated through a least absolute shrinkage and selection operator regression using an L1 penalty to improve the estimation of the shortest TC.^48^ The matrix size resulted of 53 × 53 based on the number of ICNs. We used Gaussian windows with a size of 30 TR (54 s) and a sigma of 3 TR (5.4 s) to taper along the window edges advancing 1 TR at each step, resulting in 192 windows (see Figure 1). Then, the dFNC values were Fisher's Z‐transformed and residualized with respect to significant nuisance covariates, including age, sex, education and motion (mean framewise displacement according to Power et al.^49^).

#### 2.4.1 Meta‐state analysis

In the meta‐state approach, dFNCs are modelled as the weighted sum of maximally statistically independent connectivity patterns. Each wFNC was decomposed into maximally independent prototype connectivity patterns (CPs) using a time‐independent component analysis (tICA). CP's time courses underwent a transformation in the corresponding discretized quartile so that the weight of each pattern could be either positive or negative with respect to a space state, thus being in a pro‐state or anti‐state form if positive or negative, respectively. In this manner, a time‐indexed vector of five dimensions was obtained, in which the time‐course varied from one level to another. In this framework, a subject could be in more than one meta‐state at a specific time point, and the probability of being in one meta‐state changed dynamically.^24^

To calculate the individual connectivity patterns, the meta‐state dFNC analysis was implemented through a temporal ICA (tICA), using the Infomax algorithm, with a model order of five, sufficient to include complex additive effects while maintaining a richly featured basis pattern according to the previous literature.^22^, ^24^ This analysis was repeated 10 times to obtain robust and reliable results using ICASSO.

To increase computational tractability, each value of a TC, derived from the regression of each subject dFNC information at each time window on the group of tICA connectivity patterns, was discretized into eight bins using its signed quartile, thus providing a pro‐state (positive) and an anti‐state (negative) score based on their sign, that corresponded to a specific meta‐state.^24^

For each subject, four indices were estimated to summarize the dynamic properties of the networks and to describe the trajectory of the windowed correlations among different states (see Figure 1):

- Number of states: the overall number of distinct meta‐states occupied.
- Change between states: the number of transitions from one meta‐state to another.
- State span: the maximum L1 distance (distance in the cab geometry) between meta‐states.
- Total distance: sum of L1 distances between successive meta‐states, that is, the total distance travelled by each subject along state‐space.^24^

#### 2.4.2 Cluster states analysis

The state‐based dFNC analysis was implemented through a cluster state approach to identify dFNC states in the native state space. In this case, k‐means clustering of wFNC matrices was repeated to reduce dimensionality. Each windowed functional connectivity matrix (wFNC) was defined by the value *N*(*N*−1)/2, where *N* represents the number of components of interest: K means clustering consists of a dimensional reduction technique that allows us to “collapse” the space connectivity of *N*(*N*−1)/2 > 1,000 dimensions into one, thus obtaining the most recurrent connectivity pattern: a subject will therefore be in a single specific state at any given moment.^22^

K‐means clustering allowed us to identify the four most recurrent connectivity patterns, minimizing the total variance within the most similar matrices, and obtaining the states. The number of clusters or states was determined by the elbow criterion. For each FC cluster state, the mean dwell time, defined as the average time that each subject spent in a specific state, measured as the number of consecutive windows, was estimated^22^ (see Figure 1). Cluster states that recurred in less than 10 individuals per sample were considered not reliable for group comparisons and excluded from further analysis.

### 2.5 Statistical analysis

Statistical analyses were performed using JAMOVI^50^ (https://www.jamovi.org/). Demographics, psychometric assessments, substance use patterns and brain dynamics indices were compared between samples using two‐sample *t* tests. Brain dynamics indices were also correlated with clinical and substance use parameters using Pearson's correlations. Additionally, to take into account the psychopathological differences between samples, the analyses were repeated using analyses of covariance (ANCOVA) to compare group differences and partial correlations for brain‐behaviour correlations with the score of each scale (HAM‐D, STAI‐G X1 and X2, and ADHS‐SB) as nuisance covariates, respectively. Statistical significance was assessed using a threshold of *p* = 0.05, and a false discovery rate (FDR) correction with *α* = 0.05 was used for multiple comparisons.^51^

## 3 RESULTS

### 3.1 Meta‐state analysis

**FIGURE 2:**
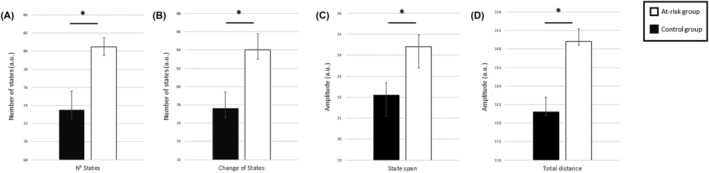
Brain dynamics estimated using meta‐states is increased in individuals at risk for CUD. The bar plots represent the magnitude of the meta‐states dynamic indices for each diagnostic group: (A) number of states, (B) changes of states, (C) state span and (D) total distance. A.U., arbitrary units. Error bars represent the standard error of the mean. *Significant difference between control and at risk subjects with a *p* < 0.05

The following brain dynamics indices were significantly increased in individuals at risk for CUD compared to controls (see Table 2 and Figure 2).

**TABLE 2:** Comparison of the meta‐state indexes between CUD at risk and controls.

	*t*	*p* ^a^
Number of states	2.35	0.021
Change between states	2.43	0.017
State span	2.72	0.008
Total distance	3.36	0.001

^a^ Results from two‐sample *t* tests.

These results were confirmed by ANCOVAs with HAM‐D, STAI‐G X1, STAI‐G X2 and ADHS‐SB (see Table 3).

**TABLE 3:** Comparisons of meta‐state dynamic indices between CUD at risk and normal controls while taking into account each individual's psychopathology using ANCOVAs with HAM‐D, STAI‐G X1, STAI‐G X2 and ADHS‐SB scores as covariates.

HAM‐D ANCOVA	*F*	*p*	STAI‐G X2 *ANCOVA*	*F*	*p*
Number of states	2.66	0.009	Number of states	2.09	0.039
Change between states	2.75	0.007	Change between states	2.21	0.030
State span	2.97	0.004	State span	2.41	0.018
Total distance	3.59	<0.001	Total distance	3.06	0.003

STAI‐G X1 ANCOVA	*F*	*p*	ADHS‐SB ANCOVA	*F*	*p*
Number of states	2.44	0.017	Number of states	1.74	0.086
Change between states	2.58	0.011	Change between states	1.77	0.080
State span	2.69	0.008	State span	2.52	0.013
Total distance	3.41	<0.001	Total distance	2.64	0.010

.

Abbreviations: ADHS‐SB, ADHS‐Selbstbeurteilungsskala; CUD, cannabis use disorder; HAM‐D, Hamilton Rating Scale for Depression; STAI‐G X1, State–Trait Anxiety Inventory German version Form X1; STAI‐G X2, State–Trait Anxiety Inventory German version Form X2.

All correlation analyses carried out between meta‐state measures and psychometric scores or consumption patterns were not significant.

### 3.2 Cluster state analysis

**FIGURE 3:**
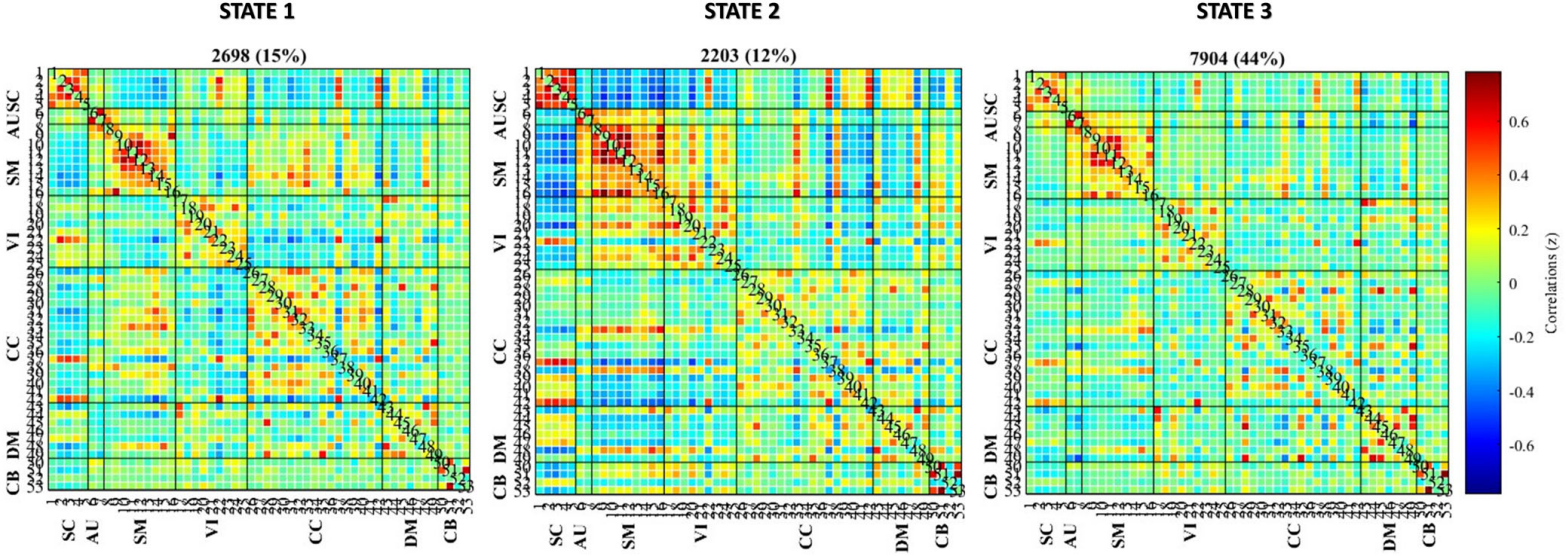
Cluster‐state structure. For each cluster state, the centroid of 53 × 53 cross‐correlation matrices was estimated using k‐means clustering and ordered by functional network domain. Percentages indicate the number of windows where a cluster state was present relative to the total number of windows in the overall sample. The colour bar indicates the magnitude of each correlation. In SC, sub‐cortical domain; AU, auditory domain; SM, sensorimotor domain; VI, visual domain; CC, cognitive‐control domain; DM, default‐mode domain; CB, cerebellar domain

Four cluster states were estimated. After discarding state four data for limited reliability (see before), three clusters were further analysed (see Figure 3).

#### 3.2.1 Cluster State 1

State 1 recurred 15% of the time. In this state, the within‐network correlations were highest in SC, SM and CC, and the between‐network correlations were highest in SC (negative with SM and CC), SM (negative with VI and SC and positive with CC) and CC (negative with VI and SC and positive with SM).

#### 3.2.2 Cluster State 2

State 2 recurred 12% of the time. In this state, within‐network correlations were highest in SC, SM and VI, and between‐network correlations were highest in SM (negative with SC, DM, some CC ICs and positive with VI), SC (negative with SM, VI and CB and positive with DM), VI (negative with SC, positive with SM), DM (positive with SC and some CC ICS, negative with SM) and CB (negative with SC).

#### 3.2.3 Cluster State 3

State 3 recurred 29% of the time. In this state, within‐network correlations were highest in SC, SM, VI, CC and DM, while between‐network correlations were reduced for SC (with AU, SM and CC), AU (with SC, SM, VIS, CC and CB), SM (with SC, AU, VIS, CC and CB), VI (with SC, AU, SM and CB), for CC (with SC, AU, SM and CB) and CB (with AU, VIS and CC).

**FIGURE 4:**
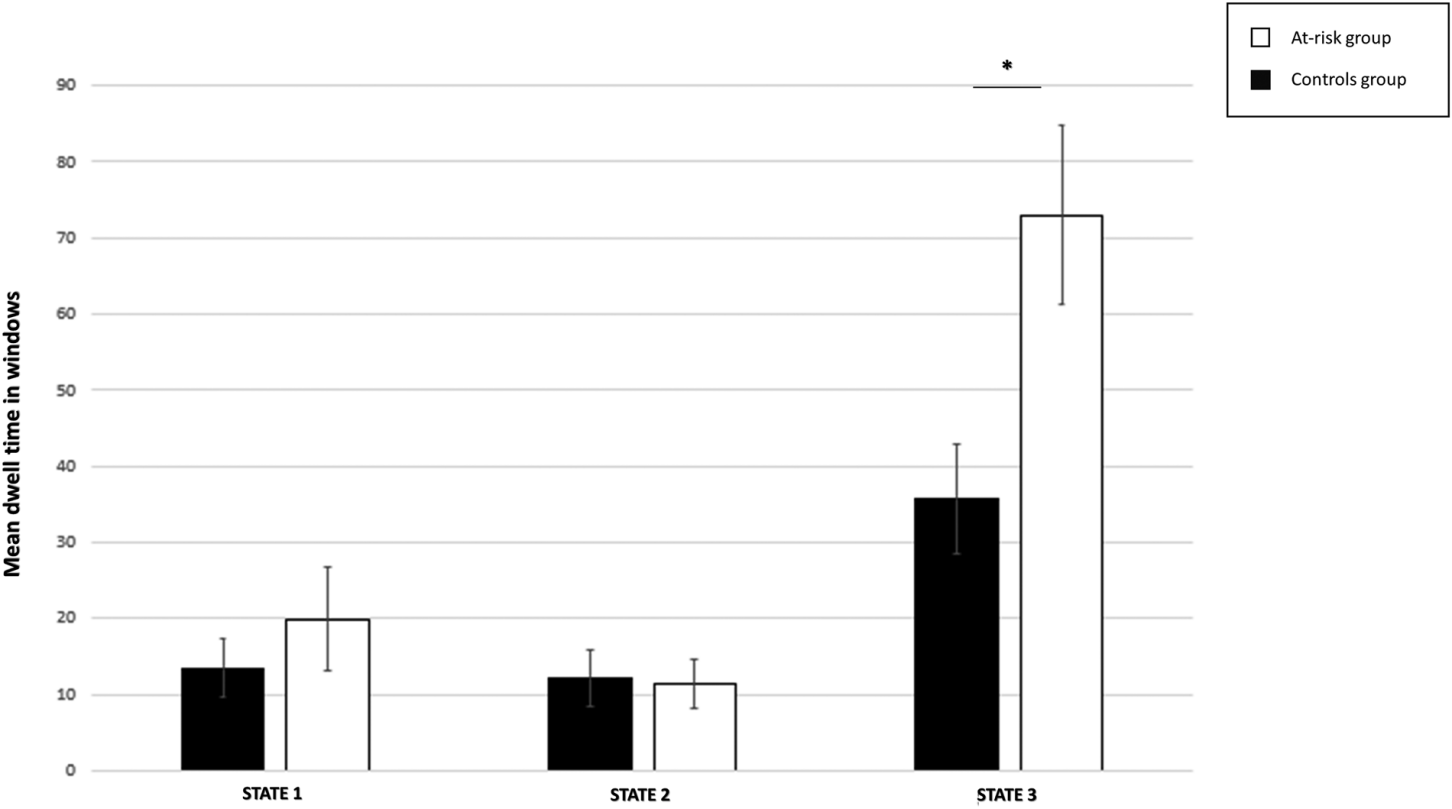
Individuals at risk for cannabis use disorder dwelled longer in State 3. The bar plot represents the mean dwell time of the three cluster states (see Figure 3 for the cluster structure). The dwell times are measured in units of repetition time (1 TR = 1.8 s). The error bars represent the standard error of the mean. *p < 0.05

When comparing the dwell time between the groups, at‐risk individuals dwelled longer only in State 3 relative to the control group (*t*_92_ = 2.85, *p* = 0.005) (see Figure 4). These results were confirmed by ANCOVAs with HAM‐D (*t*_91_ = 2.55, *p* = 0.013), STAI‐G X1 (*t*_91_ = 2.54, *p* = 0.013), STAI‐G X2 (*t*_91_ = 2.80, *p* = 0.006) or ADHS‐SB (*t*_91_ = 2.31, *p* = 0.023).

The mean dwell time in Cluster 3 and the psychometric scores or the cannabis use patterns were not significantly correlated.

## 4 DISCUSSION

The aim of this study was the identification of dFNC alterations associated with the risk of CUD. This study yielded two main findings resulting by two complimentary approaches: at‐risk individuals had a greater number of extremely volatile meta‐states and moved more frequently between them, covering a greater distance in the state space, and, at the same time within specific cluster states, they tended to stay longer in a state (Cluster State 3) with high within‐network coupling in SC, SM, VI, CC and DM, and reduced between‐network coupling.

### 4.1 Meta‐state changes in the risk of CUD

The FC of the brain networks in the risk of CUD showed increased dynamics. In particular, a higher number of meta‐states, and more frequent transitions from one meta‐state to another, reflected greater dynamic fluidity in individuals at risk for CUD relative to those at low risk. Additionally, a greater maximal L1‐distance between meta‐states and the total distance travelled in the state space indicated a greater dynamic range in at‐risk individuals. Overall, at‐risk individuals have more patterns of interrelationship between networks (meta‐states) where they persist for shorter times, and temporally subsequent states differ more than in low‐risk individuals.

Studies in neuropsychiatric disorders with cognitive impairments have shown conflicting results on dynamic functional connectivity, with some studies showing decreased values for all global brain dynamic indices in schizophrenia,^24^ autism,^52^ some forms of major neurocognitive disorder^26^ and in preterm born adolescents,^53^ and others showing higher values for these metrics, including Alzheimer's disease^54^ and other neonatal disorders such as foetal alcohol spectrum disorder.^55^

Consistent with these studies, we found a subtle cognitive impairment indicated by inattention, hyperactivity and impulsivity scales in subjects at risk for CUD. To reconcile these findings, we can hypothesize that dynamic features could be related to the subject global cognitive functioning following an inverted‐U model, so that subjects with low or high dynamic indices have impaired cognition, in the first case due to the lower possibility of configuring adaptive networks, while in the second, this is due to an instability in maintaining efficient and goal‐oriented networks with frequent changes between distant states.

### 4.2 Cluster state features in the risk of CUD

These individuals persist longer in a state characterized by higher intra‐network connectivity in the subcortical, sensorimotor, visual, default mode and cognitive‐control network domains and reduced between‐network interactions. The subcortical network domain includes the nucleus accumbens, the ventral tegmental area (VTA), the basolateral amygdala, the hippocampus, the bed nucleus of the stria terminalis, the dorsolateral striatum and the globus pallidus. THC activates presynaptic CB1 receptors (CB1R) in VTA GABAergic neurons that inhibit presynaptic GABA release, and this causes increased dopaminergic firing in the VTA, which may explain the greater intrinsic activity of this network.^56^ The subcortical regions are connected to the prefrontal regions through dopaminergic and GABAergic projections. The cognitive control network, including the prefrontal regions, plays a role in cognition, emotional regulation, salience and impulsivity that can be altered in cannabis use and dependence.^57^ Therefore, connectivity between subcortical and cognitive control networks can be crucial for the recognition of a reward in the environment and in complex affective and cognitive behaviours, including the formation and retrieval of associative and contextual fear‐ and reward‐related memories.^57^ Altered functional connectivity of the subcortical network domain has been reported in previous studies,^14^ thus suggesting that changes in dynamic connectivity may be related to altered reward processing and disinhibition. Altered dynamic connectivity of sensorimotor and visual networks supports a modulatory role of cannabis use on sensorimotor function. In this regard, it is noteworthy that recent studies have shown that individuals with high THC use have more severe neurological soft signs (NSS) compared to controls.^58^ This is probably caused by an impairment of the sensorimotor system through direct stimulation of CB1R and an alteration of dopamine signalling in the limbic/associative and sensorimotor cortical regions due to THC.^58^ Furthermore, the activity of the visual network appears to be influenced by cannabis use and is related to the development of NSS. A recent study demonstrated that chronic cannabis consumers have greater activation during a visual attention task not only in the occipital cortex but also in various frontal and parietal regions, suggesting compensatory and adaptive processes to maintain physiological network functioning.^59^ Even the default mode network, particularly in the posterior regions, has been implicated in THC dependence.^60^ Hyperactivation of this network is related to greater sensitization to drug‐seeking behaviours and decreased performance on strategic decision‐making tests,^61^, ^62^ which supports the role of these changes in the development of addiction.

Overall, our findings are in accordance with the GABA‐glutamatergic hypothesis of CUD, which posits that THC can modulate the neuronal excitability of the brain circuits by regulating GABA and glutamate signalling.^60^, ^63^ CB1Rs, which are more expressed at the level of GABAergic neurons, are activated by endocannabinoids and inhibit neurotransmitter release through retrograde synaptic transmission. GABAergic interneurons, especially fast‐spiking parvalbumin (PV)‐expressing interneurons and non‐fast spiking cholecystokinin (CCK)‐positive cells, play a substantial role in the synchronization of large numbers of glutamatergic pyramid cells. CCK‐positive cells that express CB1R work as a noise filter to remove high‐frequency signals from pyramidal cells. Endocannabinoids can determine a fine‐tuning of cortical neural oscillations by modulating these interneurons. Therefore, the disruption of endocannabinoid signalling caused by CB1R downregulation in chronic cannabis users appears to disrupt excitatory‐inhibitory balance and affects dynamic connectivity, causing a major disruption in network interaction with cortical and subcortical domains that function more independently of each other.^64^

### 4.3 Strengths and limitations

This study has some potential limitations. First, the relatively small sample size reduces the potential of detecting more subtle associations and therefore the representativeness of our findings. However, our design was sufficiently powerful to detect the moderate‐higher differences with a robust correction for multiple comparisons to avoid Type II errors. Future studies with larger sample sizes are warranted for the replication of these results.

Second, anxiety and ADHD scores were higher in at‐risk subjects relative, and this could have partially contributed to our findings. Notably, most subjects had scores below the clinical cut‐off, thus suggesting a limited impact of these variables, if any, that could be efficiently accounted for using these scores as nuisance covariates. Finally, our cross‐sectional study does not allow for the evaluation of trajectory risk.

## 5 CONCLUSION

This is the first study to evaluate dynamic connectivity in individuals at risk for CUD. We have shown that individuals at risk for CUD present, early in the course of cannabis use, a global alteration of the dynamics of brain network interplay with specific changes in subcortical and cortical circuits.

DFNC alterations can contribute to the identification of prognostic factors in individuals at risk of CUD, in whom preventive intervention can be carried out, and of novel targets for treatment. DFNC alterations could also be used as biomarkers in preclinical pharmacological trials to test drug efficacy to normalize the dFNC of at‐risk individuals' disruption to avoid their progression to addiction. Finally, longitudinal studies are needed to establish the relationship between functional connectivity dynamics and the development of cannabis use disorder to confirm their role as biomarkers of the risk.

## AUTHOR CONTRIBUTIONS

Fabio Sambataro, Robert Christian Wolf, Miriam Wittemann, Nadine D. Wolf, Wolfgang Reith, Florian Werler, Katharina M. Kubera, Vince D Calhoun and Dusan Hirjak were responsible for the study concept and design. Robert Christian Wolf, Florian Werler, Miriam Wittemann and Wolfgang Reith contributed to the acquisition of clinical and neuroimaging data. Giovanni Fazio, Daniele Olivo and Fabio Sambataro performed neuroimaging and statistical analyses and assisted with data analysis and interpretation of the findings. Giovanni Fazio and Fabio Sambataro drafted the first version of the manuscript. Robert Christian Wolf, Dusan Hirjak and Mike M. Schmitgen provided critical revision of the manuscript for important intellectual content. All authors critically reviewed the content and approved the final version for publication.

## CONFLICT OF INTEREST STATEMENT

The authors declare that no competing financial interest exists.

## ETHICS STATEMENT

The study was carried out in accordance with the Code of Ethics of the World Medical Association (Declaration of Helsinki) for experiments involving humans. All participants gave their informed written consent as approved by the Ethics Review Board of the Saarland Medical Association, Saarbrücken, Germany.

## Data Availability

The data that support the findings of this study are available from the corresponding author upon reasonable request.
